# Nanos3, a cancer-germline gene, promotes cell proliferation, migration, chemoresistance, and invasion of human glioblastoma

**DOI:** 10.1186/s12935-020-01272-1

**Published:** 2020-05-26

**Authors:** Fengyu Zhang, Ruilai Liu, Cheng Liu, Haishi Zhang, Yuan Lu

**Affiliations:** 1 Department of Laboratory Medicine, Huashan Hospital, Fudan University, 12 Wulumuqi Road, Jing-an District, Shanghai, 200040 China; 2 Department of Neurosurgery, Huashan Hospital, Fudan University, 12 Wulumuqi Road, Jing-an District, Shanghai, 200040 China

**Keywords:** Glioblastoma, Nanos3, Cancer-germline, CRISPR/Cas9, Tumorigenicity

## Abstract

**Background:**

Radiotherapy, chemotherapy, and surgery have made crucial strides in glioblastoma treatment, yet they often fail; thus, new treatment and new detection methods are needed. Aberrant expression of Nanos3 has been functionally associated with various cancers. Here, we sought to identify the clinical significance and potential mechanisms of Nanos3 in human glioblastoma.

**Methods:**

Nanos3 expression was studied in nude mouse glioblastoma tissues and glioblastoma cell lines by immunohistochemistry, Western blot, and RT-PCR. Clustered regularly interspaced short palindromic repeats (CRISPR)–Cas9 gene editing assay was performed to generate the Nanos3 knockdown glioblastoma cell lines. The effects of Nanos3 on glioblastoma cells proliferation, migration, invasion, chemoresistance, germ cell characteristics, and tumor formation were analyzed by CCK8, transwell, cell survival experiments and alkaline phosphatase staining in vitro and in nude mouse models in vivo. Correlation between the expression of stemness proteins and the expression of Nanos3 was evaluated by Western blot.

**Results:**

We found that Nanos3 was strongly expressed in both glioblastoma cell lines and tissues. Western blot and sequencing assays showed that the Nanos3 knockdown glioblastoma cell lines were established successfully, and we discovered that Nanos3 deletion reduced the proliferation, migration, and invasion of glioblastoma cells in vitro (*P *< 0.05). Nanos3 knockdown enhanced the sensitivity of glioblastoma cells to doxorubicin (DOX) and temozolomide (TMZ) (*P *< 0.05), and Nanos3^+/−^ glioblastoma cell lines did not show the characteristics of the germline cells. In addition, Nanos3 deletion inhibited subcutaneous xenograft tumor growth in vivo (*P *< 0.001). Moreover, the oncogenesis germline protein levels of CD133, Oct4, Ki67, and Dazl decreased significantly in glioblastoma cells following Nanos3 knockdown.

**Conclusions:**

Both in vitro and in vivo assays suggest that Nanos3, which is a cancer-germline gene, initiates the tumorigenesis of glioblastoma via acquiring the oncogenesis germline traits. These data demonstrate that ectopic germline traits are necessary for glioblastoma growth.

## Background

Gliomas, the most common primary brain tumor, are associated with a poor median survival time that barely exceeds 12 months despite the development of new effective treatments [1–3]. The regular care for glioblastoma patients includes surgery followed by radiation and chemotherapy; Dox and TMZ are the conventional chemotherapeutic drugs for clinical treatment of glioblastoma [4, 5]. However, Dox and TMZ are only beneficial to a subgroup of patients [1, 6]. Recurrence after regular therapy is unavoidable and ultimately results in a high mortality for glioblastoma patients [7, 8]. Tumor initiation, therapeutic resistance, recurrence, and poor prognosis have been a global problem for glioblastoma, and little is known about the genetic mechanisms involved in its pathogenesis. Moreover, unraveling the mechanism of glioblastoma pathogenesis is essential to the diagnosis, treatment, and prevention of glioblastoma.

Janic et al. [9] proposed that the acquisition of germline characteristics by somatic cells might contribute to increased fitness and survival and could contribute to the transformation of mammalian cells. Current hypotheses have defined the cancer-testis (CT) genes or cancer-germline (CG) genes, which are predominantly expressed in germline cells and have little or no expression in somatic adult tissues; however, they are aberrantly activated in various malignancies such as melanoma and other types of tumors [9, 10]. A subset of these human CG genes are suspected to contribute to oncogenesis germline traits such as immortality, invasiveness, metastasis, and hypomethylation, so they are being investigated as biomarkers [9–11]. Ectopic germline traits are essential for growth in *Drosophila* tumors expressing *malignant brain tumor* (*mbt*), and some germline genes upregulated in *mbt* tumors are orthologs of human CG genes such as NANOS1/*Nanos* [9, 12]. The upregulated germline genes in *mbt* tumors might be relevant to human cancer.

The *Nanos* genes encode a small family of evolutionarily conserved RNA-binding proteins that are required for germ cell development and embryonic patterning in diverse model organisms. The first Nanos family member described was a unique Nanos gene in *Drosophila* melanogaster, which was identified as a maternal effect gene required for abdomen formation [13]. Nanos has been widely studied and is now well known to control the differentiation of the anterior–posterior body axis, primordial germ cell (PGC) migration, maintenance of germline stem cell self-renewal and suppression of somatic cell fate during germline development [14–16]. The Nanos1 gene could maintain the testis size and promote PGC incorporation into the gonad in the male mouse [17]. In humans, three homologues of *Nanos* genes (*NANOS 1*, *2* and *3*) have been studied and have been shown to have conserved functions in germline development [14, 17, 18]. However, in oncology, it emerged that Nanos2 and Nanos3 are meiosis regulators and are dysregulated in testicular carcinoma in situ [19]. In *Drosophila*, ectopic expression of *Nanos* drives the growth of malignant brain tumors, such as glioblastoma [9]. Upregulation of NANOS1 and NANOS3 facilitates the oncogenic growth of p-Rb-deficient cells, suggesting that *Nanos* has a dynamic role in cancer cell proliferation [20]. However, the role of Nanos3 in human glioblastoma is still unknown.

Bone morphogenetic proteins (BMPs), which are embryonic proteins, are considered potent inhibitors of glioblastoma during development and clonogenicity [21, 22]. It has been demonstrated that BMP signals can induce glioblastoma cells differentiation and attenuate tumorigenic phenotype in vitro as well as in vivo [22–25]. CD133 was introduced as a cancer stem cell (CST) marker [26], and had been involved in the tumorigenesis of different cancers [27]. Oct4 was a transcription factor of the POU family that played an important role in self-renewal and maintenance of pluripotency in embryonic stem cells, and is also considered as a promising CST marker [27, 28]. Both CD133 and Oct4 are identified as glioblastoma stem/progenitor cell marker [29] and have been involved in the tumorigenesis of glioblastoma [27]. In this regard, we evaluated whether Nanos3 regulate the expression of CD133 and Oct4 in human glioblastoma. *Deleted in azoospermia like* (Dazl) served as CG gene [30] and stem cell marker [31–33], could participate in early proliferation, differentiation, and maintenance of male and female germ cells [31–33]. An important issue arising from the above is whether these germline cells-associated genes are re-expressed in human glioblastoma. To estimate whether there is a relationship between Nanos3 and the tumorigenesis of glioblastoma, we used CRISPR/Cas9 gene-editing technology to build glioblastoma Nanos3^+/−^ cell lines and evaluated whether knockdown of Nanos3 could inhibit tumor growth, migration, invasion, and resistance. In addition, we explored the potential molecular mechanisms linking Nanos3 and the cancer-germline gene in human glioblastoma cells.

## Methods

### Cell lines and reagents

The GBM cell lines A172 and U251 were purchased from the Institute of Fudan IBS Cell Center (HNC241, HNC1088, FDCC, Shanghai, China), and the human glioblastoma LN229 cell line was kindly provided by Guoxiang Jin (the First Affiliated Hospital, Army Medical University). Normal human astrocytes (NHA) were bought from the KeyGEN Biotech Company (KG578, Nanjing, China). All cells were cultured at 37 °C in 5% CO_2_ in Dulbecco’s modified Eagle medium (DMEM, HyClone) containing 10% (v/v) fetal bovine serum, 4 mM glutamine, 100 IU/ml penicillin, 100 µg/ml streptomycin and 1% nonessential amino acids (Thermo, Carlsbad, CA, USA). Antibodies against Cas9 (Abcam, ab204448), Nanos3 (Abcam, ab70001), cyclin D1 (Abcam, ab40754), Gapdh (Abcam, ab37168) and α-tubulin (Abcam, ab7291) were purchased from Abcam (Cambridge, UK). The Cell Counting Kit-8 (CCK-8) reagent (CK04) was purchased from DOJINDO Molecular Technologies, Inc. (Japan). An Alkaline Phosphatase Stain Kit (SK-5300) was purchased from Vector Laboratories, Inc. (Burlingame, CA, USA). Puromycin dihydrochloride (60210ES25) was purchased from Yeasen Biotech Co., Ltd. (Shanghai, China). Blasticidin S hydrochloride (15205), Doxorubicin hydrochloride (DOX) (D1515), DMH2 (SML 1535), and BMP4 (B2680) were purchased from Sigma Aldrich (St. Louis, MO, USA), and temozolomide (TMZ) was purchased from Merck (T2577, Darmstadt, Germany).

### Immunofluorescence assay

A172, U251, and LN229 cells were used for immunofluorescence analyses. Cells were seeded at a density of 10,000 cells/cm^2^ on glass coverslips pretreated with 0.1 mg/ml poly-l-lysine (C0313, Beyotime) to promote adherence. After 24 h, the slices were then washed twice with phosphate-buffered saline (PBS) and fixed with 4% paraformaldehyde (PFA) for 20 min at room temperature. To stain cytoplasmic markers, slices were permeabilized by incubation with 0.3% Triton-X-100 for 5 min at room temperature. Cells were blocked by incubation with 5% bovine serum albumin (BSA, Sigma, USA) for 1 h at room temperature and then incubated overnight at 4 °C with the primary antibodies. Controls were performed with 5% BSA as the primary antibody. After washing with PBS, the cells were incubated for 1 h at RT with an AlexaFluor 488 anti-rabbit IgG secondary antibody (1:2000; Invitrogen, CA, USA). Cell nuclei were stained with DAPI (0.5 μg/ml; Beyotime, China). Coverslips were examined with an inverted fluorescence microscope (Olympus, Germany). All the antibodies used for immunofluorescence staining are shown above.

### Western blotting

Total protein was lysed with RIPA lysis buffer (P0013B, Beyotime, China) and phenylmethanesulfonyl fluoride (PMSF, 1 mM, ST506, Beyotime, China) cocktails; then, they were separated by 12% sodium dodecyl sulfate-polyacrylamide gel electrophoresis and transferred to a polyvinylidene fluoride membrane (Millipore, Bedford, UK). The membrane was blocked for 2 h at room temperature and then incubated with primary antibody overnight at 4 °C. The membrane was then incubated with the HRP-conjugated secondary antibody (CST, USA) for 1 h. The immunoconjugates were detected with an enhanced chemiluminescence substrate (Thermo, USA) using a chemiluminescence imaging system (Tanon, Shanghai, China). Band density was analyzed with ImageJ software. The antibodies used for detecting protein expression are shown above.

### Immunohistochemistry

Tissue samples were fixed in 10% formalin, paraffin-embedded and cut into 3 μm sections, which were mounted on superfrost plus microscope slides and dried at 60 °C for 1 h. Tissues were deparaffinized by incubating in xylene and rehydrated in an ethanol gradient with decreasing amounts of ethanol until the final wash, which was water. Sodium citrate-hydrochloric acid buffer (pH 6.0, C8532, Sigma, USA) was used for antigen retrieval, and a 3% H_2_O_2_ solution was applied to quench endogenous peroxidase activity. The slides were then blocked in 10% goat serum (ab7481, Abcam, USA) for 1 h. The slides were incubated with the appropriate primary antibodies overnight at 4 °C. After incubation with the primary antibody, all slides were incubated with appropriate HRP secondary antibodies and stained with a DAB kit (ab64238, Abcam, USA) and with hematoxylin solution (MHS1, Sigma, USA). Finally, slides were dehydrated, cleared and mounted with a permanent mounting medium. Images were acquired under a microscope (Leica, Germany).

### RNA isolation and RT-PCR

Total RNA was extracted with Trizol (15596018, Thermo, USA) according to the manufacturer’s instructions, and the concentration of RNA was detected by a NanoDrop2000 spectrophotometer (Thermo, USA). Total RNA (500 ng) was reverse-transcribed using a PrimeScript™ RT reagent kit (RR036, Takara, Japan). Quantitative RT-PCR was performed using SYBR premix (RR820, Takara, Japan) according to the manufacturer’s instructions. qPCR experiments were run, and the melting curves of the amplified products were used to determine the specificity of amplification. The threshold cycle number for the genes analyzed was normalized to human GAPDH. All reactions were performed in triplicate, and the results were analyzed via the 2^−ΔΔCt^ method. The primers used for detecting gene expression were human Nanos3-F: CATTTATTGAGGGCTGACTGGAT; human Nanos3-R: CGGAACTCCTGTGCTTTGTCT; human GAPDH-F: TGCACCACCAACTGCTTAGC; human GAPDH-R: GGCATGGACTGTGGTCATGAG.

### Cell proliferation assay

GBM cells were seeded in 96-well plates at a density of 1 × 10^3^ cells per well, and then, they were incubated for 0, 1, 2, 3, 4, 5, 6 and 7 days. Then, 100 µl of reagent comprising 90 µl DMEM and 10 µl of CCK-8 solution was added to each well, and the plates were incubated for 2 h at 37 °C. The absorbance was measured at 450 nm with a microplate reader (Biotek, USA).

### Alkaline phosphatase staining

For alkaline phosphatase (AP) staining, GBM WT cells and Nanos3 deletion cells were treated with a VECTASTAIN ABC-AP kit (Vector Laboratories, USA) according to the manufacturer’s instruction. After AP staining, 10 microscopic fields (20× magnification) of each treatment were randomly selected, and AP-positive colonies were counted.

### CRISPR/Cas9‐mediated *Nanos3* knockdown

CRISPR/Cas9‐mediated *Nanos3* Knockdown in GBM cells was carried out following the protocol of Ran et al. [34]. To generate Nanos3-Knockdown cells using CRISPR–Cas9 gene editing, two different short guide RNAs (sgRNAs) against NANOS3 were purchased from Sigma (HS5000010259; HS5000010260). The Nanos3-sgRNA sequence is as follows: GGCGAAGGCTCAGACTTCCCGG; GTGGACATGGAGGGAGAGCAGG. The *Nanos3* gene was cloned into a CRISPR/Cas 9 vector: hU6-gRNA-PGK-Puro-T2A-BFP. The lenti-cas9 pSpCas9 (BB)‐2A‐GFP (PX458) plasmid was a gift from Feng Zhang (Addgene plasmid #48138). Lenti-Cas9 was transfected into 70–80% confluent GBM cells using X-tremeGENE 9 DNA Transfection Reagent (6365787001, Sigma-Aldrich, USA) and lenti-090 and lenti-091. Puromycin selection was performed for 3–5 days with a final concentration of 1 µg/ml, 3 µg/ml and 2 µg/ml in A172, U251, and LN229 cells, respectively; Lenti-*Nanos3* sgRNA-treated cells were then seeded, and another 3–5 days of blasticidin selection was performed using a final concentration of 7 µg/ml, 22 µg/ml, 10 µg/ml in A172, U251, and LN229 cells, respectively. Later, a double selection of clones growing in blasticidin-containing medium and expressing Blue Fluorescent Protein (BFP) was performed. Positive clones were isolated and transferred to 6-well plates; finally, the positive clone was verified by sequencing. Nanos3 deletion was further verified by Western blotting of lysate from the stable cells.

### In vivo experiments: xenograft model

All animal studies were performed according to the “Guide for the Care and Use of Laboratory Animals” of the National Institutes of Health. GBM cells (1.5 × 10^5^) stably expressing Nanos3^+/−^ and GBM cells expressing Nanos3^+/+^ were subcutaneously injected into 4-week-old female BALB/c nude mice (Shanghai Lab. Animal Research Center, China). Tumor growth was assessed by measuring tumor diameters with Vernier calipers once every 6 days. Tumor volumes were calculated according to the equation: tumor volume (mm^3^) = (length × width × width)/2. The survival of the remaining mice was assessed via Kaplan–Meier analysis. When the experiments were stopped, experimental animals were sacrificed by carbon dioxide inhalation, and tumors were removed for biochemical (frozen tissue) and histological (paraffin fixed tissue) analyses.

### Statistical analysis

Exact representations of error bars are indicated in each figure. All experiments were executed in triplicate unless otherwise specified. Statistical analysis was performed via GraphPad Prism version 6.0 (San Diego, CA, USA). Differences between groups were considered statistically significant when *P *< 0.05.

## Results

### Nanos3 expression is upregulated in both glioblastoma cell lines and glioblastoma tissues

**Fig. 1:**
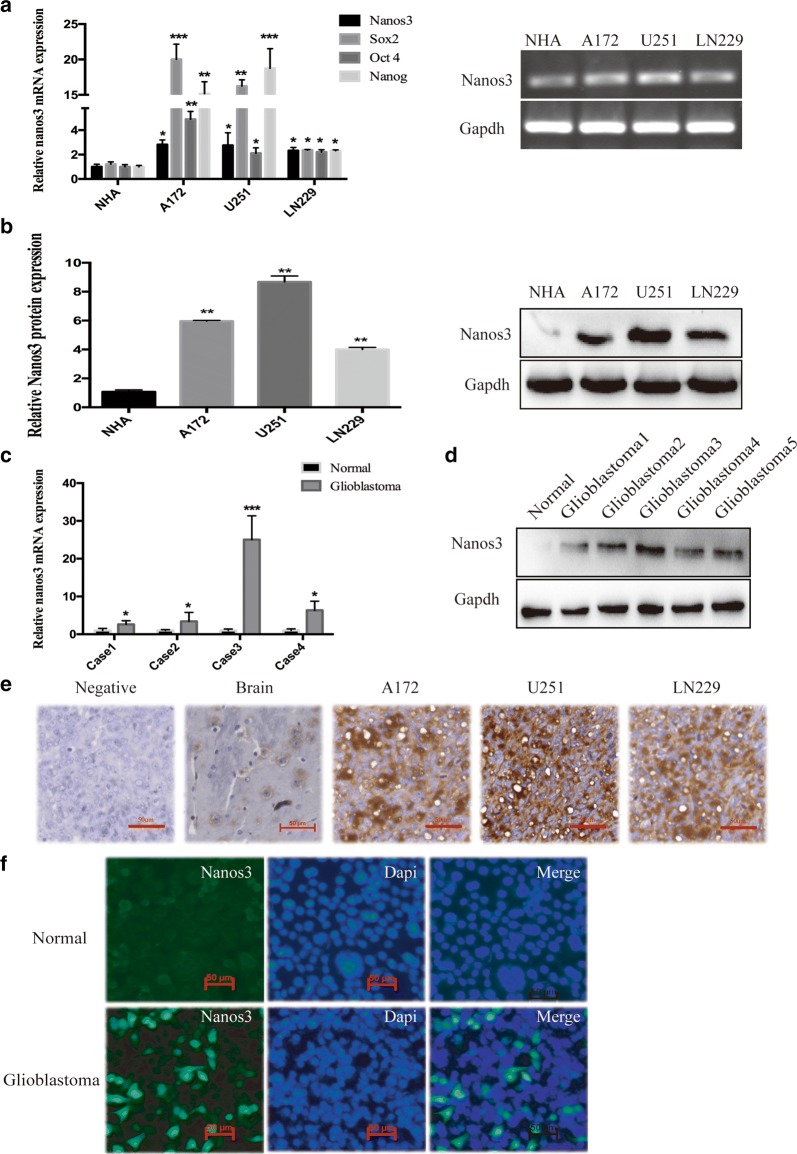
Expression of Nanos3 in glioblastoma tissues and glioblastoma cell lines. **a** Whole-cell RNA extracts were further prepared from NHA cells and the following three GBM cell lines: A172, U251, and LN229. The Nanos3 RNA level was determined by RT-PCR analysis. **b** Whole-cell proteins were extracted from NHA cells and the GBM cell lines A172, U251, and LN229. The Nanos3 protein level was examined by western blot analysis. **c** RT-PCR analysis determined Nanos3 expression in normal mouse tissues and tumor tissues of the xenograft tumor models. **d** Western blot analysis determined Nanos3 protein expression in normal brain tissues and tumor tissues of the xenograft tumor models. **e** Immunohistochemistry data shows Nanos3 expression in normal HMC3 cells, normal brain tissue and GBM cells (A172, U251, and LN229 cells). **f** Immunofluorescence shows Nanos3 expression in NHA and A172 cells. All experiments were carried out in triplicate. Data are shown as the mean ± SE. *P < 0.05, **P < 0.01, ***P < 0.001

To evaluate whether cancer-germline genes are upregulated in human glioblastoma, RT-PCR experiments, Western blot assays and immunohistochemistry staining were performed to detect the expression of germline-associated genes, namely, Nanos3, in human glioblastoma cell lines and glioblastoma tissues (Fig. 1). The glioblastoma tissues were obtained from the implanted tumor xenografts of glioblastoma multiforme (GBM) cells in mice. RT-PCR showed that Nanos3 was generally overexpressed in GBM cells and the mouse glioblastoma tissues (case 1–4) compared with its expression in normal human astrocytes (NHA) cells and the normal mouse brain (Fig. 1a, c). Moreover, compared with NHA cells, the RNA expression of Sox2, Oct4, and Nanog were increased in GBM cells. Western blot data confirmed that Nanos3 was overexpressed in all GBM cell lines and mouse glioblastoma tissues (glioblastoma 1–5) compared with NHA cells and normal mouse brains (Fig. 1b, d). Immunohistochemical analysis revealed that Nanos3 was frequently expressed in glioblastoma tissues implanted from the GBM cell lines (A172, U251, and LN229 cells), and the expression of Nanos3 was higher than it was in the normal brain (Fig. 1e). Immunofluorescence indicated that Nanos3 was expressed in the glioblastoma cells, especially in A172 cells, but not expressed in the NHA cells (Fig. 1f, Additional file 1: Figure S1). Thus, Nanos3 is expressed at high levels in glioblastoma cells and glioblastoma tissues.

### CRISPR/Cas9-mediated knockdown of Nanos3 in GBM cell lines

**Fig. 2:**
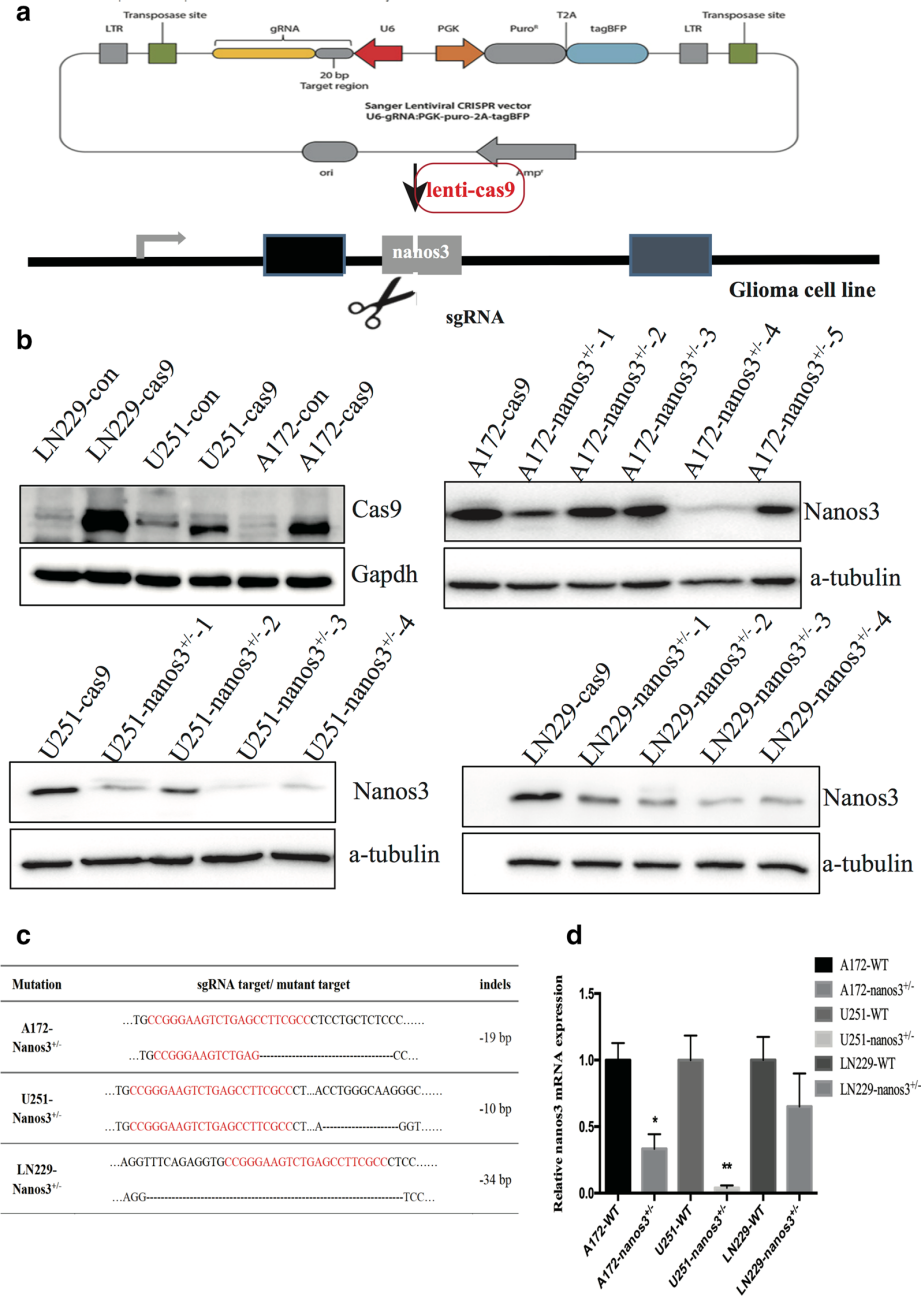
CRISPR/Cas9-mediated knockdown of Nanos3 in GBM cell lines. **a** Lenti-sgRNA against Nanos3 and lenti-Cas9 plasmids were transfected into GBM cell lines (A172, U251, and LN229). A schematic overview of the procedure used for the CRISPR/Cas 9 knockdown experiment is shown. **b** Western blot analysis detected whether cas9 protein was transfected into GBM cells successfully and whether Nanos3 protein was deleted. **c** The PCR band for the Nanos3-sgRNA infected cells was subcloned and analyzed by sanger sequencing. The sequences of three candidate clones are shown. **d** RT-PCR assays were utilized to test the RNA expression of the three candidate clones

To explore the function of Nanos3 in glioblastoma genesis, we first generated a Nanos3 single knockdown clone in GBM cell lines via the CRISPR/Cas9 system. CRISPR/Cas9 technology has been confirmed to be an effective gene-editing tool that can target an individual gene, cause an in-frame reading error and subsequently induce a transcriptional-level disruption of the gene. Hence, we selected 1 site to target in a Nanos3 exon. Lenti-Nanos3-sgRNA was transfected into A172, U251 and LN229 Nanos3 WT cells, separately, 5 days after lenti-Cas9 to test the efficiency of gene-editing (shown in Fig. 2a). Then, the expression of green and blue fluorescence was observed by fluorescence microscopy to detect the transient transfection rate (figure not shown). Single cells were isolated from pools harvested 5–7 days after transfection following common culture conditions. Western blotting revealed that glioblastoma cells were successfully transfected with cas9 (Fig. 2b), and the cells co-transfected with lenti-Cas9 and lenti-sgRNA had significantly inhibited Nanos3 expression (Fig. 2b). Furthermore, Sanger sequencing was used to determine whether the target gene was edited (Fig. 2c). Finally, the expression of Nanos3 RNA in the Nanos3 deletion cells was also significantly lower than it was in the Nanos3 WT cells (Fig. 2d). For the homozygous mutants, Nanos3^−/−^ could inhibit cell proliferation and could not grow in the culture conditions. All of the deletion cell lines were heterozygous (Nanos3^+/−^), but the expression of Nanos3 was still reduced at both the protein and RNA levels in these cells (Fig. 2b, d).

### Knockdown of Nanos3 inhibits glioblastoma cell growth and migration in vitro

**Fig. 3:**
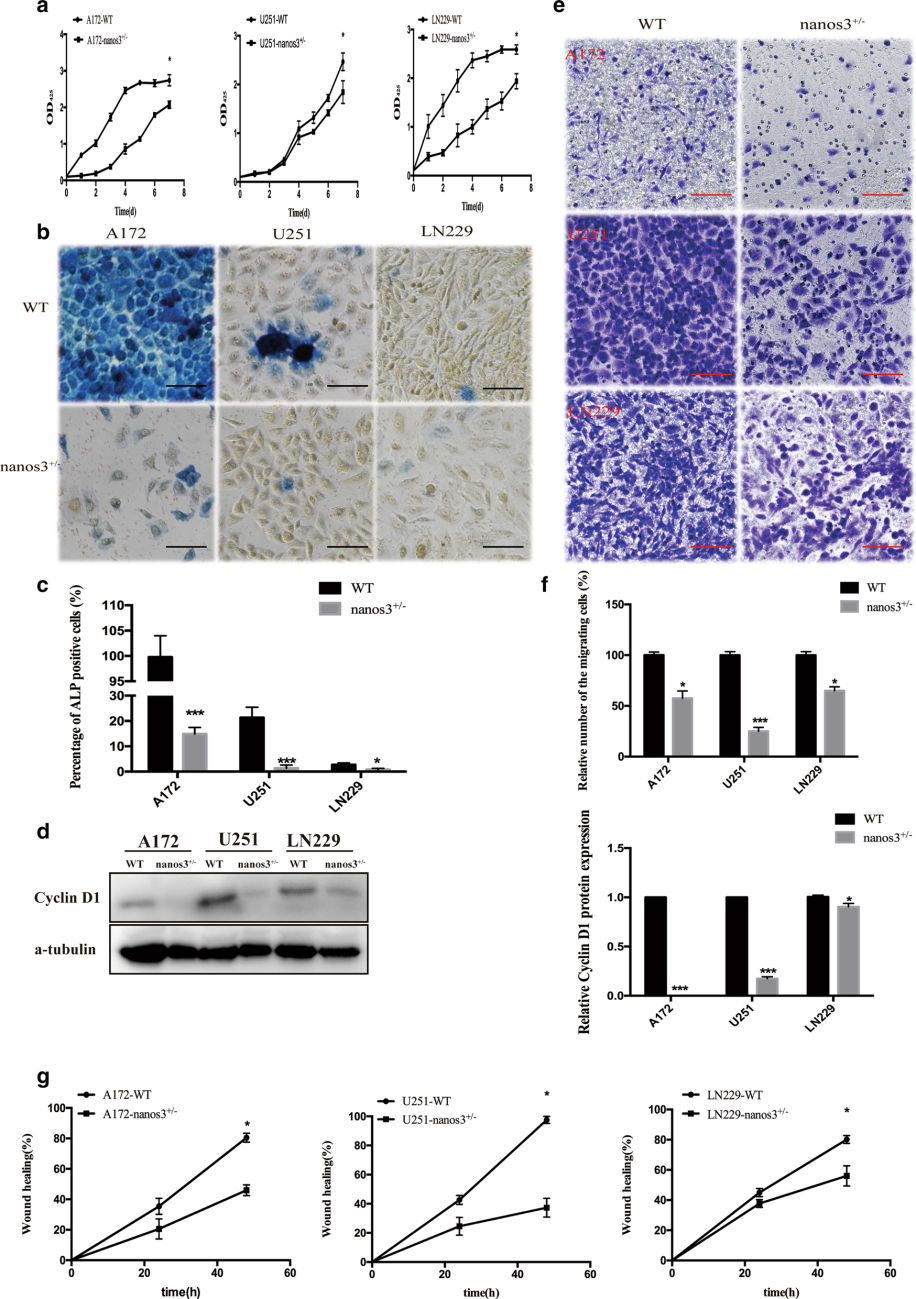
Knockdown of Nanos3 inhibits glioblastoma cell growth and migration in vitro. **a** A CCK-8 cell proliferation assay was performed after Nanos3 deletion in A172, U251, and LN229 cells. **b** An alkaline phosphatase stain assay was performed between the WT GBM cell lines and the Nanos3 deletion cells. Images were taken from the inverted microscope (bars = 50 μm; magnification ×200). **c** The statistical analysis of the positive ALP staining of the glioblastoma cells. **d** Western blot analysis determined Cyclin D1 protein expression in GBM cell lines and the nanos3 KD cells. The statistical analysis of the protein expression in the right pane. **e** Cell migration assays were performed after Nanos3 deletion in A172, U251, and LN229 cells. A172, U251, and LN229 cells with Nanos3 knockdown exhibited decreased ability to migrate through the Boyden chamber compared with the WT GMB cell lines (bars = 50 μm; magnification ×200). **f** The statistical analysis of the ability of the glioma cells’ migration. **g** The statistical analysis of the wound-healing assay. The wound-healing assay was performed between GBM WT cells and Nanos3 knockdown cells. All experiments were carried out in triplicate. All data are shown as the mean ± SE. *P < 0.01, **P < 0.01, ***P < 0.001

Nanos3 promotes germline cell growth during the development of germline cells, and it plays a crucial role in brain tumor growth in *D. melanogaster.* To further investigate whether Nanos3 knockdown could inhibit cell proliferation, we performed cell counts with CCK-8 assays. The results of the CCK-8 cell proliferation assays showed that Nanos3 knockdown significantly inhibited the proliferation of A172, U251 and LN229 cells (Fig. 3a), and the proportion of G1 phase cells increased significantly, and that of G2 phase cells decreased in Nanos3 KD cells (Additional file 1: Figure S5). These results indicated that knockdown of Nanos3 could induce the G1 phase cell cycle arrest in GBM cells, and thus Nanos3 affected glioblastoma cells proliferation pattern. Nanos3 deletion also reduced the germline characteristics of the glioblastoma cells, as shown by the AP stain, and loss of germline characteristics might lead the GBM cells to not be easily oncogenic (Fig. 3b, c). Because cell proliferation is directly connected to cell cycle and cyclin D1 acts as a key regulator of the cell cycle [35]. Then we detected whether Nanos3 regulated Cyclin D1 expression using western blot assay, and found that inhibition of Nanos3 reduced the expression of Cyclin D1 (Fig. 3d). To investigate the role of Nanos3 in A172, U251, and LN229 cells in migration, wound healing and transwell migration assays were performed. We found that the number of Nanos3^+/−^ cells was decreased in contrast to the Nanos3 WT cells in migration experiments (Fig. 3e, f). The scratch experiments also revealed that wound recovery was markedly decreased in Nanos3 deletion cell lines, separately, in A172, U251, and LN229 cells (Fig. 3g). These results showed that the silencing of Nanos3 inhibited the migration ability of A172, U251, and LN229 cell lines.

### Knockdown of Nanos3 inhibits glioblastoma cell chemoresistance and invasion in vitro

**Fig. 4:**
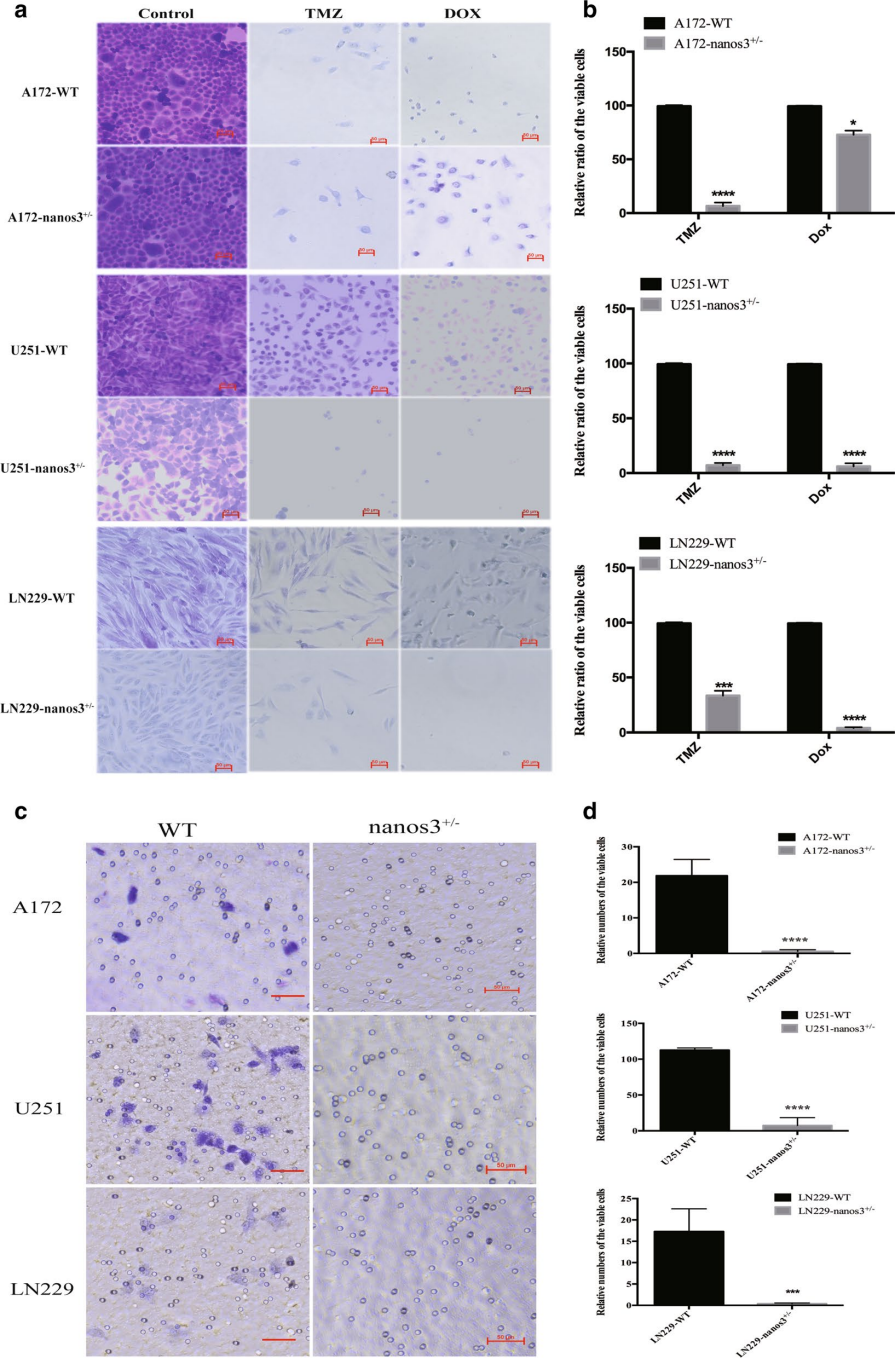
Knockdown of Nanos3 inhibits glioblastoma cell chemoresistance and invasion in vitro. **a** Nanos3 KD cells were significantly more sensitive to DOX and TMZ than GBM WT cells (A172, U251, and LN229). Viable cells were stained with crystal violet, images were taken with an inverted microscope (bars = 50 μm; magnification ×100). **b** The statistical analysis of the glioma cell resistance. All experiments were carried out in triplicate. Data are shown as the mean ± SE. ^#,^*P < 0.05; ^##,^**P < 0.01; ^###,^***P < 0.001. **c** A matrigel cell invasion assay was performed after the deletion of Nanos3 in GBM cell lines. Nanos3^+/−^ cells showed a decreased ability to invade through the Matrigel chamber compared with the Nanos3 WT GMB cell lines. Cells were stained with crystal violet, images were taken using an inverted microscope (magnification ×100). **d** The statistical analysis of the glioma cell invasion. All experiments were carried out in triplicate. Data are shown as the mean ± SE. ***P < 0.001

To investigate the fundamental reasons for the chemoresistance to therapy in glioblastoma, we explored whether Nanos3 deletion inhibited the chemoresistance of GBM cells to DOX and TMZ. Upon treatment with DOX and TMZ, we found that knocking-down Nanos3 resulted in significantly lower survival of A172, U251, and LN229 cells (Fig. 4a, b). Together, the assays suggested that Nanos3 may inhibit therapeutic resistance during glioblastoma treatment. To investigate the function of Nanos3 in A172, U251, and LN229 cell invasion, the transwell Matrigel invasion assays were utilized. The transwell invasion assay results showed that fewer Nanos3 knockdown cells had passed through the Matrigel-coated chambers in comparison to WT cells (Fig. 4c, d). These results revealed that the silencing of Nanos3 inhibited the invasiveness of GBM cell lines.

### Nanos3 inhibits the formation of glioblastoma via inhibiting the stemness of glioblastoma cells

**Fig. 5:**
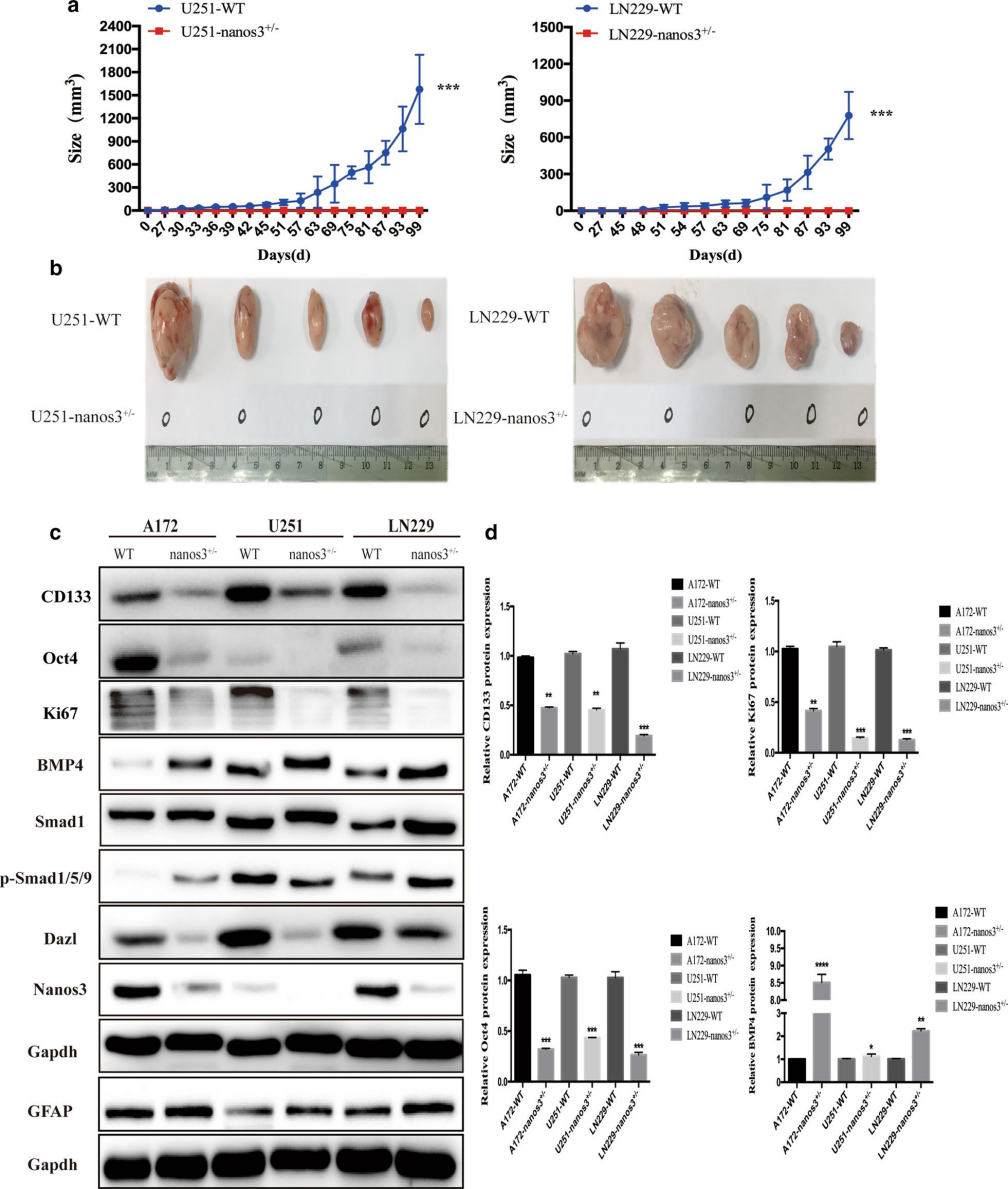
Nanos3 inhibits the formation of glioblastoma via inhibiting the stemness of glioblastoma cells. **a** The tumor growth sizes were recorded every 6 days between Nanos3 WT and Nanos3^+/−^ GBM cell lines in xenograft tumor models; **b** Tumor growth was observed from GBM cells with Nanos3 alterations that were implanted subcutaneously in nude mice. n = 5; data are shown as the mean ± SE. ***P < 0.001. **c** Western blot analysis of the relative protein levels of CD133, Oct4, Ki67, Dazl, BMP4, and GFAP in Nanos3^+/−^ and WT GBM cells. **d** Quantitative analysis of the relative protein levels of CD133, Oct4, Ki67 and BMP4 in GBM cells was carried out in triplicate. Data are shown as the mean ± SE. *P < 0.05, **P < 0.01, ***P < 0.001

To test for the function of Nanos3 in vivo, GBM cell lines and Nanos3^+/−^ cells were inoculated subcutaneously into the backs of immunocompromised nude mice. Injection of approximately 10^5^ GBM cells showed a rapid tumor growth in vivo (Fig. 5a, b), whereas no tumor growth was observed in mice injected with Nanos3^+/−^ GBM cells. GBM cells with homogenous Nanos3 deletion were unable to initiate tumorigenesis, and recipient mice remained viable after > 6 months. In this work, to examine whether Nanos3 could participate in the process of stemness and proliferation, we examined the expression of the stem cells markers CD133, Oct4 and Dazl, which are found to be upregulated in GBM cell lines compared to NHA cells in the mRNA level (Fig. 1a, b). BMPs are considered potent inhibitors of glioblastoma during development, and BMP treatment can decrease the tumorigenicity of glioblastoma in vivo [22, 36–38], BMPs negatively regulate stemness and induce differentiation via activation of the Smad signaling cascade [21]. Subsequently, we detected the protein expression of BMP4 and astrocytic differentiation marker glial acidic fibrillary protein (GFAP) using western blot assay, and found that Nanos3 knockdown could increase the protein expression of BMP4 and GFAP, but not the expression of Smad1, which acts in complex with the phosphorylated versions of Smad1, Smad5, and Smad9; then, this complex entered the nucleus to control the transcription of target genes. Blocking the BMP pathway using a BMP receptor kinase inhibitor (DMH2) did not induce Nanos3 to participate in the BMP pathway. In contrast, exogenous activation of BMP signaling using BMP4 did not contribute to the activation of BMP signaling by Nanos3 in the GBM cells (Additional file 1: Figure S3b). We then investigated whether Nanos3 knockdown could affect the stemness and proliferation of glioblastoma. Western blot assays showed that the knockdown of Nanos3 significantly decreased the protein expression of CD133, Oct4, Dazl, and Ki67 in these glioblastoma cell lines but did not alter the expression of β-catenin (Additional file 1: Figure S3a). Therefore, Nanos3 knockdown was found to suppress the oncogenicity of glioblastoma by inhibiting the cancer-germline characteristics and tumor cells growth, and increasing the glioma cells differentiation (Fig. 5c, d).

## Discussion

Nanos was originally discovered and researched in *Drosophila melanogaster* (fruit fly) [39]. The *Nanos* gene was primarily found to be crucial for anterior–posterior axis polarity, abdomen formation, and germ cell development [39–41]. The RNA-binding protein Nanos3 is highly conserved in the germline stem cell function, were it works with other Nanos orthologs, such as Nanos2 and Nanos3 in *Mus musculus* [17]. Since Old et al. [11] proposed an embryonic rest theory for the origin of cancer, cancer cells and germ cells have been found to share several characteristics, such as rapid proliferation, migration, and colonization. *Nanos* genes are responsible for germline traits such as survival, which are also important for tumor cells [42]. There is plentiful evidence for the ectopic activation of germline genes during the progression of several human cancer types [43]. CRISPR/Cas9 technology is a powerful method which was widely used in tumorigenesis [44, 45] and metastasis [46] as well as genes associated with drug response [47–49]. In this work, we built the Nanos3^+/−^ GBM cell lines via CRISPR/Cas9 knockout technology and the Nanos3^+/−^ GBM cell lines grew stably for generation. Then, we demonstrated that ectopic expression of the germline gene Nanos3 in human glioblastoma brain tumors and its association with tumorigenicity. Moreover, high-level ectopic expression of Nanos3 is an independent marker of overall survival and may therefore be essential for the progression of glioblastoma. Our results confirm this view and show the close similarity between tumor formation and tissue development. Interestingly, Nanos3 is expressed in PGCs at mouse E7.25, and loss of function results in a lack of germ cells and a failure of PGCs fails to migrate into the gonad [17]. Nanos protein expression has also been linked to increased cell migration, invasion and cell survival [16, 50]. We also found that Nanos3 deletion could significantly inhibit glioblastoma cell proliferation, migration, and invasion. These results are consistent with the fact that Nanos3 modulates essential aspects of human germ cell development during the cell cycle [14]. It is worth noting that Nanos3 is strongly expressed in adult germ cells but not in adult somatic tissues, except the brain; however, Nanos3 is ectopically expressed in many human cancers. This expression pattern has been illustrated by naming these genes ‘cancer-germline’ genes [51, 52], and these findings are in agreement with the previous study of Julaton et al. [14, 16]. In *D. melanogaster*, *Nanos* was found to be one of the crucial germline genes that were upregulated in a brain tumor model [9]. These results demonstrate that Nanos3 may be considered a ‘cancer-germline’ gene involved in the tumorigenicity of glioblastoma.

Glioblastoma, the most common neurological tumor, could be inhibited by BMP treatment. Differentiation-inducing properties of BMPs could inhibit the tumorigenicity of GBM cells, making BMPs promising candidates for GBM therapy [22, 53–55]. In this work, Nanos3 knockdown enhances the protein expression of BMP4, which in turn activates the astrocyte’s fate switch, in agreement with the differentiation potential of BMPs in GBM cells. To further investigate whether Nanos3 enhances the tumorigenicity through BMP/Smads signaling pathway, western blot analysis showed that the presence of BMP4 significantly reduced the expression of Nanos3, and DMH2 could restore the expression level of the Nanos3 in GBM cell lines. p-Smad1/5/9 are the complex proteins in the downstream of BMP signaling pathway. BMP4 could induce small mothers against decapentaplegic 1/5/9 (Smad1/5/9) phosphorylation. It is reported that BMP4 can inhibit the glioblastoma cell growth. A172 could inhibit the protein expression of BMP4 and BMP signaling pathway, but U251and LN229 could only specifically inhibit the expression of BMP protein, not the entire pathway. These might be related to the different expression of p53 genotype in different GBM cells, since A172 expressed the p53 WT gene, but U251 and LN229 expressed the p53 mutation. Interventions to modulate BMP signaling have the potential to enhance the effect of conventional chemotherapy dependent on the mutational status of p53 [56]. Subsequently, we tested whether Nanos3 could participate in the WNT signaling pathway using western blot assay, and showed that there were no difference in β-Catenin expression (Additional file 1: Figure S3A). It is suggested that Nanos only inhibited stemness gene expression in glioma but did not participate in other signaling pathways to regulate glioma formation. These results suggest that Nanos3 inhibits BMP protein and suppresses the GBM cells differentiation but not participate in the BMP/Smads pathway. Besides, the expression of glioblastoma stem/progenitor cell markers like CD133 and Oct4 have also been widely investigated in vitro and in vivo concerning their tumor-initiating potential, migratory and proliferative capacity and resistance to chemotherapy [29, 57]. Dazl is the maker of the germline cells, nanos3 knockdown could significantly reduce the germline trait. We found that knockdown of Nanos3 significantly reduced GBM cell proliferation, tumorigenicity, and the characteristics of cancer-germline cells. It is speculated that Nanos3 may induce the oncogenesis of glioblastoma via simulating the characteristics of embryonic germ cells. Therefore, *Nanos3* gene could represent ideal candidates, since it belongs to a unique group of CG genes that primarily contribute to some key process of tumor development, including unlimited proliferation, metastasis, adaption to cellular energetics constraints, and resistance to apoptosis. The observation that Nanos3 gene exerts oncogenic functions may explain the origin of the tumor cells. Further researches on these CG genes may therefore open the way to the development of highly selective anti-cancer therapies.

## Conclusion

In summary, our results strongly suggest that Nanos3 may play a crucial role in glioblastoma development and progression. Nanos3, which is a cancer-germline gene, influences glioblastoma progression by enhancing cell proliferation, migration, and invasion. This implies that human cancer-germline genes are suspected to contribute to oncogenesis germline traits such as immortality, migration, invasiveness, and survival, suggesting that Nanos3 may function as an important marker of cancer in the germline and the detection of glioblastoma.

## Supplementary information

**Additional file 1: Figure S1.** Expression of Nanos3 in glioblastoma cell lines. **Figure S2.** Colony formation of GBM cells after chemotherapy. **Figure S3.** The relationship between Nanos3 and BMP pathway. **Figure S4.** The regulatory relationship between Nanos3 and Dazl. **Figure S5.** Effect of Nanos3 on glioblastoma cell cycle.

## Data Availability

The datasets generated during the current study are applicable.

## Abbreviations

CG: Cancer-germline
MBT: Malignant brain tumor
PGC: Primordial germ cell
CRISPR: Clustered regularly interspaced short palindromic repeats
sgRNAs: Single guide RNAs
GBM: Glioblastoma multiforme
NHA: Normal human astrocytes
AP: Alkaline phosphatase
DOX: Doxorubicin
TMZ: Temozolomide
KD: Knockdown
WT: Wild type
BMP: Bone morphogenetic proteins
PBS: Phosphate-buffered saline
CCK-8: Cell Counting Kit-8
BSA: Bovine serum albumin
RT: Room temperature
BFP: Blue Fluorescent Protein
DMEM: Dulbecco’s modified Eagle medium
