# Supplementary material for: Nanos3, a cancer-germline gene, promotes cell proliferation, migration, chemoresistance, and invasion of human glioblastoma
Source: Cancer Cell Int. 2020 May 26;20:197. doi: 10.1186/s12935-020-01272-1 (PMC7249350; doi:10.1186/s12935-020-01272-1)
Supplement: Supplementary file 1 — Additional file 1: Figure S1. Expression of Nanos3 in glioblastoma cell lines. Figure S2. Colony formation of GBM cells after chemotherapy. Figure S3. The relationship between Nanos3 and BMP pathway. Figure S4. The regulatory relationship between Nanos3 and Dazl. Figure S5. Effect of Nanos3 on glioblastoma cell cycle. [file 12935_2020_1272_MOESM1_ESM.docx]

**Nanos3, a cancer-germline gene, promotes cell proliferation, migration, chemoresistance and invasion of human glioblastoma**

Fengyu Zhang^1#^, Ruilai Liu^1#^, Cheng Liu^1^, Haishi Zhang^2*^, Yuan Lu^1*^

**Additional Figure legends**

**
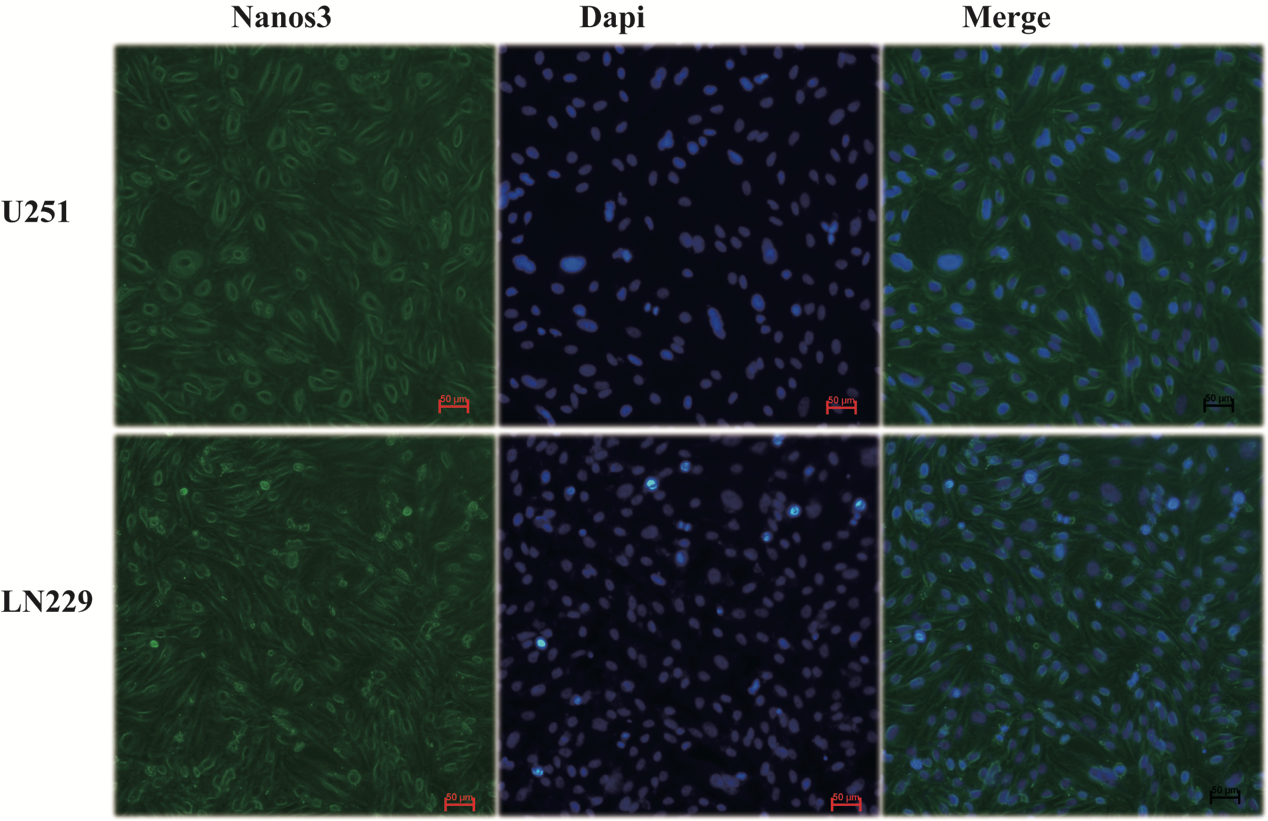
**

**Figure S1. Expression of Nanos3 in glioblastoma cell lines**

Immunofluorescence detects the expression Nanos3 in U251 and LN229, bars = 50 um

**
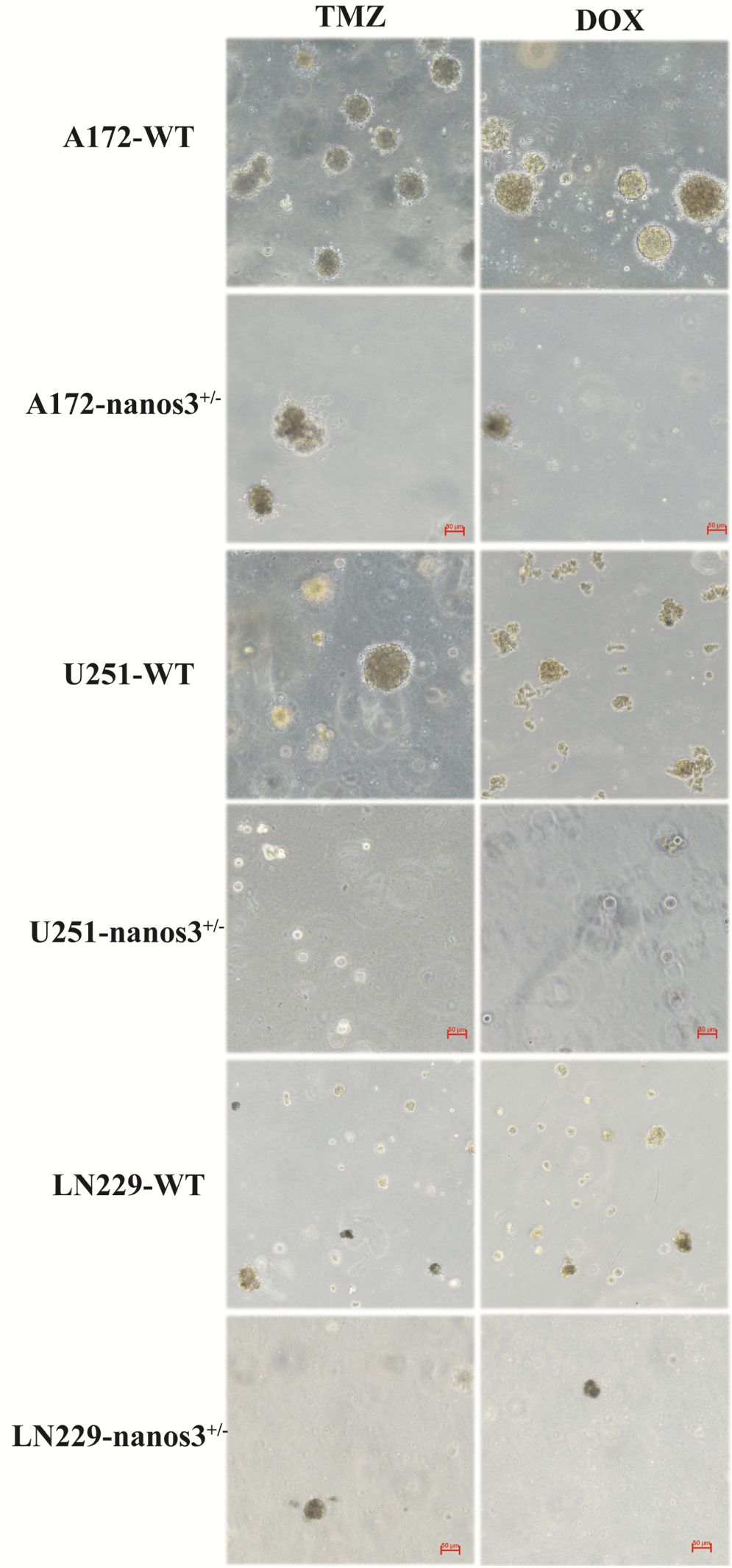
**

**Figure S2. Colony formation of GBM cells after chemotherapy**

Nanos3 KD cells were significantly more sensitive to DOX and TMZ than GBM WT cells (A172, U251, and LN229). Colony formation of GBM WT cells and GBM Nanos3 KD cells after chemotherapy, images were taken with an inverted microscope (bars = 50 μm; magnification × 100).

**Figure S3. The relationship between Nanos3 and BMP pathway.**

**a** Western blot analysis of protein expression in GBM WT and Nanos3 knockdown cell lines. **b** A172 cells and A172 Nanos3^+/-^ cells were treated with DMSO, BMP4 (20 ng/ml), and DMH2 (2 uM) for 24h, and whole cell lysis were extracted. The proteins from the BMP pathway were examined by western blot analysis.


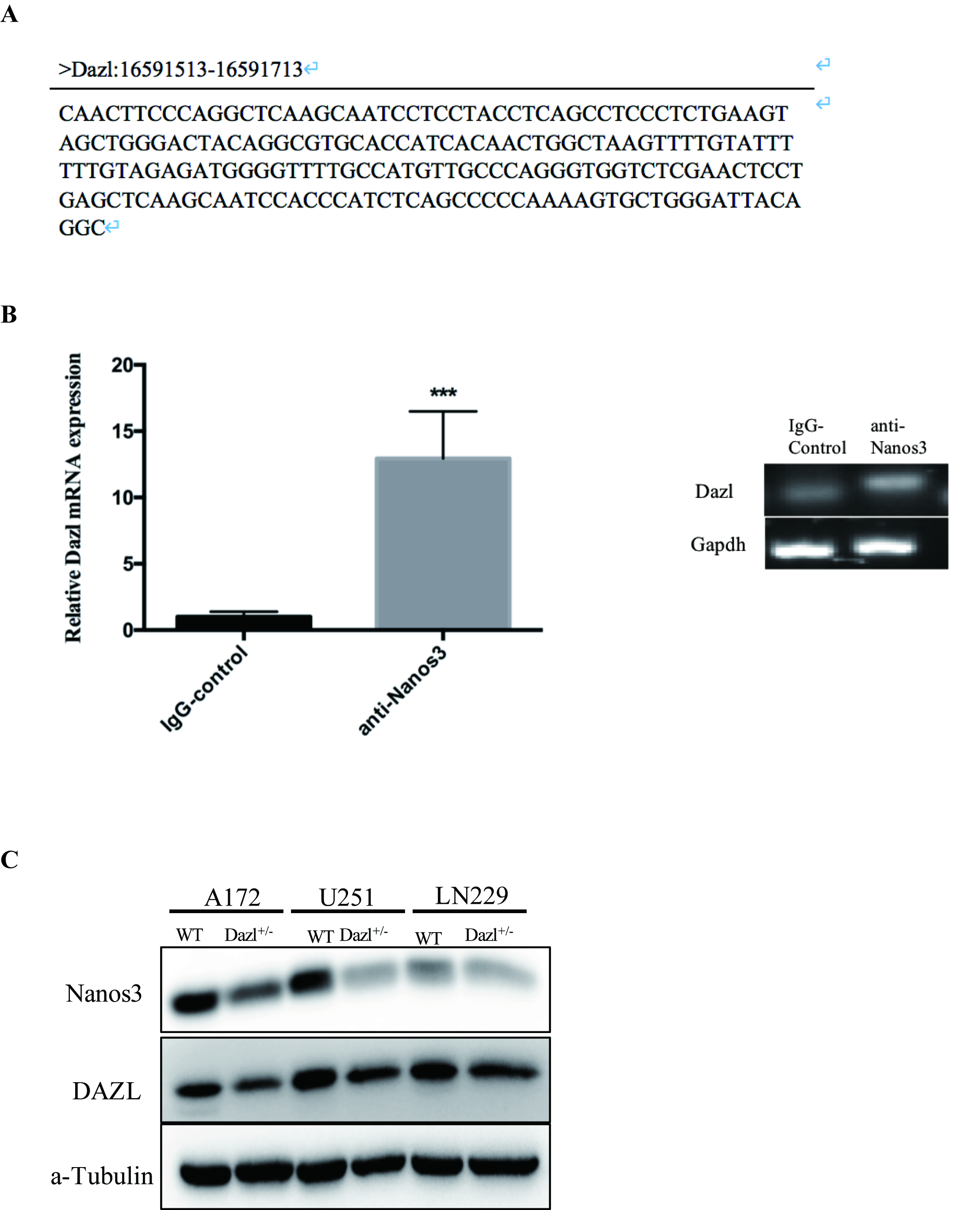


**Figure S4. The regulatory relationship between Nanos3 and Dazl.**

**a** RIP-Seq technology showed the downstream targeting RNA sequence of Nanos3 in GBM cell lines. **b** qRT-PCR assay identified the downstream targeting RNA of Nanos3 in GBM cell lines. **c** Western blot showed the protein expression of Nanos3 in GBM WT cells and GBM Dazl^+/-^ cells.


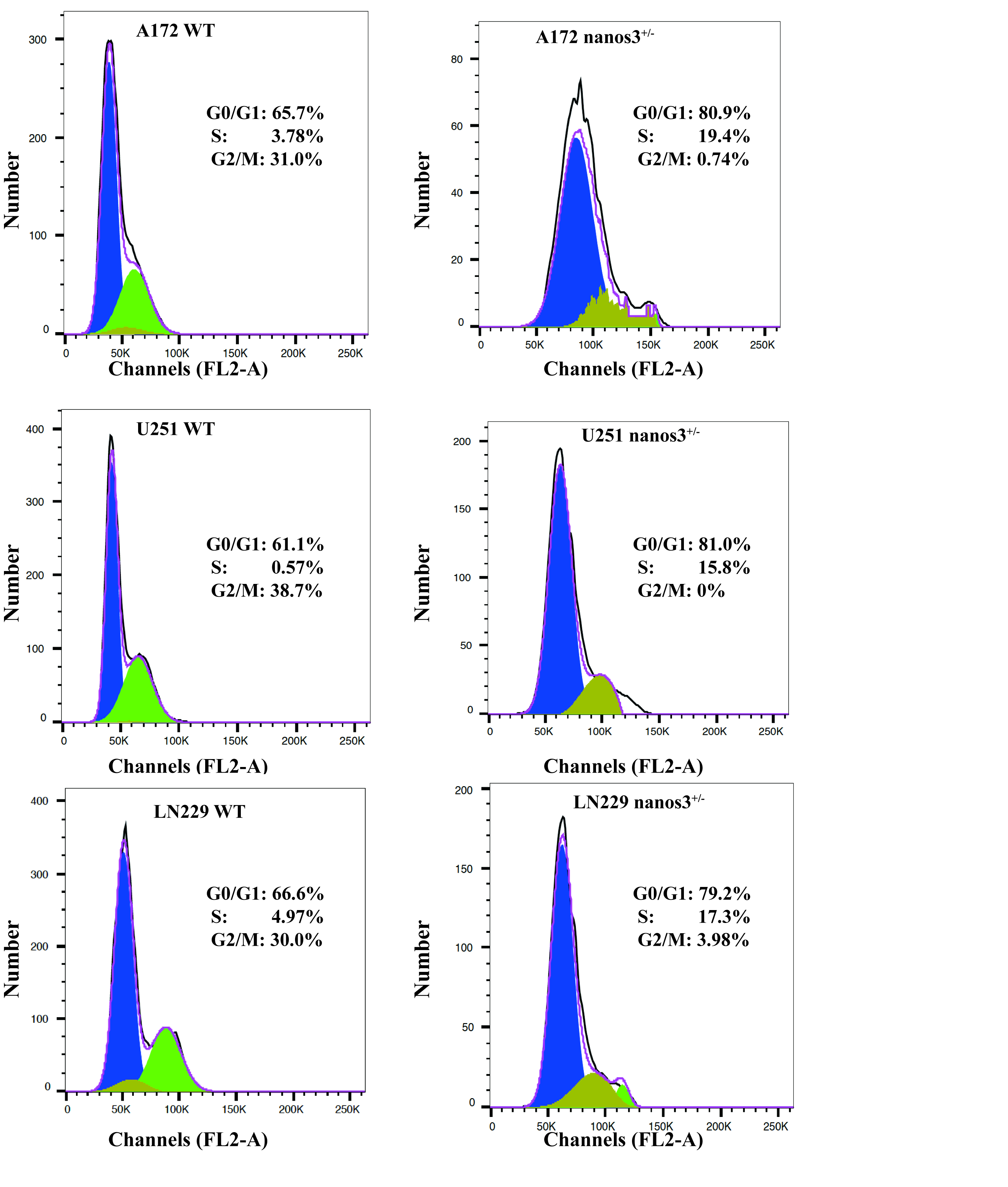


**Figure S5. Effect of Nanos3 on glioblastoma cell cycle.**

FACS analysis showed the effect of Nanos3 on different cell cycle in GBM cell lines.
